# Supplementary material for: The gut microbiota in the common kestrel (Falco tinnunculus): a report from the Beijing Raptor Rescue Center
Source: PeerJ. 2020 Dec 1;8:e9970. doi: 10.7717/peerj.9970 (PMC7718788; doi:10.7717/peerj.9970)
Supplement: Table S1 [file peerj-08-9970-s001.docx]

| **Sample Name** | **Collection Date** |
| --- | --- |
| E1 | 2019/6/23 |
| E2 | 2019/6/26 |
| E3 | 2019/6/29 |
| E4 | 2019/7/2 |
| E5 | 2019/7/4 |
| E6 | 2019/7/6 |
| E7 | 2019/7/11 |
| E8 | 2019/7/13 |
| E9 | 2019/7/16 |
