# Supplementary material for: The gut microbiota in the common kestrel (Falco tinnunculus): a report from the Beijing Raptor Rescue Center
Source: PeerJ. 2020 Dec 1;8:e9970. doi: 10.7717/peerj.9970 (PMC7718788; doi:10.7717/peerj.9970)
Supplement: Table S2 [file peerj-08-9970-s002.docx]

| **BAR** | bright, alert, responsive | **m** | mouse |
| --- | --- | --- | --- |
| **Iso** | isoflurane | **PO** | per os (given orally) |
| **KA** | kind of small cage | **R** | right |
| **LRS** | lactated ringer’s solution | **SQ** | subcutaneously |
| **NOLO** | nothing leftovers | **SSD** | silver sulfadiazine cream |
| **TMT** | tarsometatarsus | **D1(2,3,4)** | digit 1(2,3,4) |
