# Supplementary material for: The gut microbiota in the common kestrel (Falco tinnunculus): a report from the Beijing Raptor Rescue Center
Source: PeerJ. 2020 Dec 1;8:e9970. doi: 10.7717/peerj.9970 (PMC7718788; doi:10.7717/peerj.9970)
Supplement: Table S3 [file peerj-08-9970-s003.docx]

| **2019-06-22** |
| --- |
| **Weight: 176 grams** BAR, stood in box when arrived.  All feathers were oiled and IB. Some feathers on head were missing. A small wound on R TMT, R foot was swollen. Bandaged it with SSD.  SQ 10 ml LRS. PO 4 ml LRS. Put in KA, gave 2 small m with organ exposed. |
|  |
| **2019-06-25** |
| BAR, stood on perch in KA. NOLO, gave 35g m.  Treatment under anesthesia: mask, Oxy 0.8, ISO 2%. R foot was swollen seriously. Wound was dry on R TMT, debrided the dead tissue until bleeding. Bone and tendon were exposed. flushed a lot by iodine, then bandaged with SSD. |
|  |
| **2019-06-28** |
| BAR, stood in KA. NOLO, gave 20g m with Vit +Ca with meds below in: Amoxi-Clav: 0.60 ml (31.25 mg/ml susp) Meloxicam: 0.06 ml (1.5 mg/ml susp)  16:30 ate all. gave 17g m with meds below in: Amoxi-Clav: 0.60 ml (31.25 mg/ml susp) Meloxicam: 0.06 ml (1.5 mg/ml susp) |
|  |
| **2019-07-01** |
| **Weight: 210 grams** BAR, Stood on perch. Just eat a mouse's head. weight with bandage SQ 6 ml LRS Amoxi-Clav: 0.60 ml (31.25 mg/ml susp) PO Meloxicam: 0.06 ml (1.5 mg/ml susp) PO  16:20 gave 1 m(22g) with meds in Amoxi-Clav: 0.60 ml (31.25 mg/ml susp)  Meloxicam: 0.06 ml (1.5 mg/ml susp) |
|  |
| **2019-07-04** |
| BAR, stood on high perch, NOLO.  Treatment under anesthesia: mask, Oxy 1.0, ISO 2-3% Wound on R TMT looked well, clean and moist. R foot was less swollen now. Flushed it a lot with iodine and bandaged with SSD. Did ball bandaged of R foot. Vocalized a lot during handling.  SQ 10 ml LRS. PO meds below: Amoxi-Clav: 0.60 ml (31.25 mg/ml susp)  Meloxicam: 0.06 ml (1.5 mg/ml susp)  17:00 gave 33g m with meds in. Amoxi-Clav: 0.60 ml (31.25 mg/ml susp)  Meloxicam: 0.06 ml (1.5 mg/ml susp) |
|  |
| **2019-07-05** |
| BAR, stood on high perch. LO 1/2 m. Gave 16g m with meds in. Amoxi-Clav: 0.60 ml (31.25 mg/ml susp)  Meloxicam: 0.06 ml (1.5 mg/ml susp)  17:00 gave 18g m with meds in. Amoxi-Clav: 0.60 ml (31.25 mg/ml susp) Meloxicam: 0.06 ml (1.5 mg/ml susp) |
|  |
| **2019-07-10** |
| **Weight: 220 grams** BAR, NOLO.  Gave 27g m + Vit + Ca. |
|  |
| **2019-07-13** |
| BAR, NOLO, gave 26g m.   Treatment under anesthesia: mask, Oxy 1.0, ISO 2-3% Wound on R TMT looked well, clean and moist. RD1 tendon was exposed, the digit has no function. Flushed it a lot with iodine and bandaged with SSD. Did ball bandaged of R foot. |
|  |
| **2019-07-15** |
| **Weight: 214 grams**  BAR, NOLO.  Gave 27 g m + Vit + Ca. |
