# Supplementary material for: The gut microbiota in the common kestrel (Falco tinnunculus): a report from the Beijing Raptor Rescue Center
Source: PeerJ. 2020 Dec 1;8:e9970. doi: 10.7717/peerj.9970 (PMC7718788; doi:10.7717/peerj.9970)
Supplement: Table S4 [file peerj-08-9970-s004.docx]

| **Sample** | **Total Sequence** | **Total Base** |
| --- | --- | --- |
| E1 | 37,752 | 16,171,011 |
| E2 | 43,125 | 18,329,625 |
| E3 | 36,514 | 15,460,836 |
| E4 | 47,372 | 19,503,837 |
| E5 | 51,133 | 21,774,581 |
| E7 | 38,005 | 15,755,419 |
| E6 | 45,343 | 19,307,557 |
| E8 | 47,496 | 19,882,483 |
| E9 | 42,734 | 18,149,609 |
