# Supplementary material for: The gut microbiota in the common kestrel (Falco tinnunculus): a report from the Beijing Raptor Rescue Center
Source: PeerJ. 2020 Dec 1;8:e9970. doi: 10.7717/peerj.9970 (PMC7718788; doi:10.7717/peerj.9970)
Supplement: Table S5 [file peerj-08-9970-s005.docx]

| **Sample** | **OTU No.** | **Species No.** | **Genus No.** | **Family No.** | **Order No.** | **Class No.** | **Phylum No.** |
| --- | --- | --- | --- | --- | --- | --- | --- |
| E1 | 66 | 63 | 51 | 35 | 22 | 12 | 8 |
| E2 | 649 | 477 | 341 | 206 | 128 | 50 | 22 |
| E3 | 515 | 339 | 249 | 133 | 84 | 34 | 18 |
| E4 | 578 | 471 | 346 | 182 | 101 | 36 | 19 |
| E5 | 235 | 205 | 158 | 80 | 49 | 21 | 12 |
| E6 | 476 | 370 | 269 | 147 | 88 | 40 | 19 |
| E7 | 263 | 217 | 169 | 90 | 58 | 23 | 15 |
| E8 | 317 | 266 | 198 | 104 | 60 | 29 | 16 |
| E9 | 317 | 268 | 211 | 120 | 70 | 33 | 19 |
