# Supplementary figures and images for: The gut microbiota in the common kestrel (Falco tinnunculus): a report from the Beijing Raptor Rescue Center
Source: PeerJ. 2020 Dec 1;8:e9970. doi: 10.7717/peerj.9970 (PMC7718788; doi:10.7717/peerj.9970)

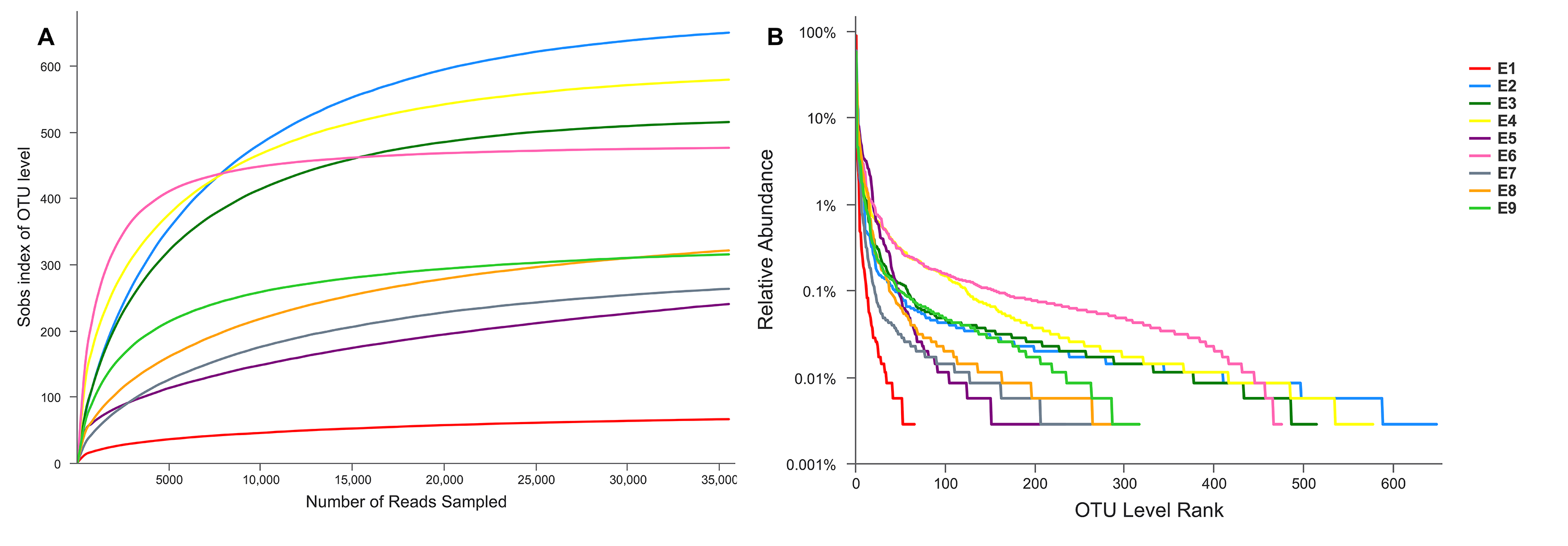

Supplement: Figure S1 — Rank Abundance Curves (B) reflect the species richness by the span of curves onthehorizontal axis. While the evenness of the bacterial communities was depicted by the shape of curves. [file peerj-08-9970-s006.png]
